# Enhanced stability and linearly polarized emission from CsPbI_3_ perovskite nanoplatelets through A-site cation engineering

**DOI:** 10.1038/s41377-025-02135-y

**Published:** 2026-01-02

**Authors:** Woo Hyeon Jeong, Junzhi Ye, Jongbeom Kim, Rui Xu, Xinyu Shen, Chia-Yu Chang, Eilidh L. Quinn, Hyungju Ahn, Myoung Hoon Song, Peter D. Nellist, Henry J. Snaith, Yunwei Zhang, Bo Ram Lee, Robert L. Z. Hoye

**Affiliations:** 1 School of Advanced Materials Science and Engineering, Sungkyunkwan University, Suwon, Republic of Korea; 2 Inorganic Chemistry Laboratory, University of Oxford, Oxford, UK; 3 Department of Materials Science and Engineering, Ulsan National Institute of Science and Technology (UNIST), Ulsan, Republic of Korea; 4 School of Physics, Sun Yat-sen University, Guangzhou, China; 5 Clarendon Laboratory, Department of Physics, University of Oxford, Oxford, UK; 6 Pohang Accelerator Laboratory (PAL) Pohang, Gyeongbuk, Republic of Korea; 7 Department of Materials, University of Oxford, Oxford, UK

**Keywords:** Nanoparticles, Quantum dots

## Abstract

The anisotropy of perovskite nanoplatelets (PeNPLs) opens up many opportunities in optoelectronics, including enabling the emission of linearly polarized light. But the limited stability of PeNPLs is a pressing challenge, especially for red-emitting CsPbI_3_. Herein, we address this limitation by alloying formamidinium (FA) into the perovskite cuboctahedral site. Unlike Cs/FA alloying in bulk thin films or nanocubes, FA incorporation in nanoplatelets requires meticulous control over the reaction conditions, given that nanoplatelets are obtained in kinetically-driven growth regimes instead of thermodynamically-driven conditions. Through in-situ photoluminescence (PL) measurements, we find that excess FA leads to uncontrolled growth, where phase impurities and nanoplatelets of multiple thicknesses co-exist. Restricting the FA content to up to 25% Cs substitution enables monodisperse PeNPLs, and increases the PL quantum yield (from 53% to 61%), exciton lifetime (from 18 ns to 27 ns), and stability in ambient air (from ~2 days to >7 days) compared to CsPbI_3_. This arises due to hydrogen bonding between FA and the oleate and oleylammonium ligands, anchoring them to the surface to improve optoelectronic properties and stability. The reduction in non-radiative recombination, improvement in the nanoplatelet aspect ratio, and higher ligand density lead to FA-containing PeNPLs more effectively forming edge-up superlattices, enhancing the PL degree of linear polarization from 5.1% (CsPbI_3_) to 9.4% (Cs_0.75_FA_0.25_PbI_3_). These fundamental insights show how the stability limitations of PeNPLs could be addressed, and these materials grown more precisely to improve their performance as polarized light emitters, critical for utilizing them in next-generation display, bioimaging, and communications applications.

## Abstract (web summary)

Alloying formamidinium into the A-site of red-emitting CsPbI_3_ perovskite nanoplatelets enhances their long-term stability and production of linearly polarized light through improved superlattice formation, offering a pathway towards reliable polarized light sources.

## Graphical abstract

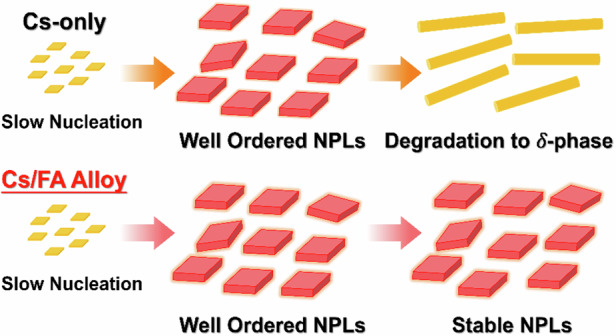

## Introduction

Colloidal lead halide perovskite nanocrystals (PNCs) have emerged as one of the most promising materials candidates for display applications, owing to their narrow spectral bandwidth, high defect tolerance, and exceptional luminescence properties, such as near unity photoluminescence quantum yield (PLQY)^1–8^. Moreover, the bandgap of PNCs can easily be tuned by varying their composition and size due to quantum and dielectric confinement. The ability to precisely control the synthesis of these materials enables the shape and size of these nanocrystals to be tailored across different dimensionalities: 0D quantum dots, 1D nanorods, 2D nanoplatelets, and 3D nanocubes^9–11^. Among these, perovskite nanoplatelets (PeNPLs), which are a few unit cells thick and strongly confined only in the out-of-plane direction, exhibit appealing properties, including high exciton binding energy and exciton fine structure splitting^6^. Exciton fine structure splitting enables the production of linearly polarized light, making these PeNPLs excellent candidates for optical applications requiring polarization, for example, to enhance the efficiency and contrast from light-emitting diode (LED) displays. Other benefits from linearly polarized light include improving directionality in lasers, improved resolution in bioimaging, enabling optical encoding in anti-counterfeit labels, enhancing selectivity in photodetectors, and providing polarization control in quantum light sources^12–18^.

Despite these excellent optoelectronic properties, there remain important challenges with PeNPLs. One of these is maintaining high PeNPL monodispersity in colloidal solution or after depositing to form thin films, which is usually adversely affected by aggregation or surface-reconstruction-induced merging. Achieving monodisperse red-emitting iodide-based PeNPLs with uniform monolayer thickness remains an important challenge due to thermodynamic instability caused by the larger ionic radius of iodine anions^19^. This undesirable behavior can broaden the emission profile, or lead to multiple distinct peaks from electronically-isolated PeNPLs with different thicknesses^9,20^. The phase and conformational transitions present in CsPbI_3_ further limit their stability. The black phases (*α*, *β*, and *γ*) of CsPbI_3_, which are intrinsically photoactive, are less stable at room temperature compared to the yellow *δ*-phase^21–25^. The ionic radius of the Cs^+^ cation is insufficiently large to ideally fit in the cuboctahedral sites in the cubic perovskite structure, such that there is a facile phase transition to *δ*-CsPbI_3_^26,27^.

Recently, a variety of strategies have been reported to improve the stability and optical properties of CsPbI_3_ PeNPLs. Organic ligands, such as ammonium halides and phosphonic acids, have been used to control the synthesis and passivation of PeNPLs, demonstrating the formation of well-aligned CsPbI_3_ PeNPLs^28,29^. Similarly, divalent metal cation dopants (e.g., Mn²⁺, Zn²⁺) have been incorporated into CsPbI_3_ PeNPLs to enhance structural stability and achieve well-ordered, low-aspect-ratio PeNPLs^19,27,30^. However, although these approaches have enhanced the colloidal stability of red-emitting PeNPLs, the fundamental issue of achieving phase-stable NCs in thin film form in air remains unresolved, leading to phase transitions occurring in a few days^27,28,30^. In the case of PNCs, various strategies such as ligand engineering^22,23,31^, metal ion doping^32,33^, and core–shell structure formation using inorganic shells^34,35^ have been explored to improve the extrinsic stability of the CsPbI_3_ perovskite structure. Another approach that has been adopted to improve the intrinsic structural stability of bulk thin films and single crystals of CsPbI_3_ is by incorporating formamidinium (FA⁺) cations, which have larger ionic radii than Cs⁺. This A-site cation (Cs and FA) alloying has been shown to stabilize the perovskite structure while suppressing optical losses in PNCs^36–43^. However, for PeNPLs, direct synthesis strategies for A-site cation alloys have not yet been reported, as PeNPL synthesis is more sensitive and demands more meticulous control over reaction conditions than PNCs. Achieving structural stabilization of red-emissive PeNPLs via A-site alloying requires a deeper understanding of the synthesis mechanisms, growth processes, and specific properties to successfully develop PeNPLs as promising emitters.

Herein, we rationalize how FA alloying into the A-site of CsPbI_3_ influences the growth kinetics, and how this in turn influences the shape, uniformity, and self-assembly of the nanoplatelets, as well as how FA incorporation affects the structural and environmental stability of the PeNPLs. We use in-situ photoluminescence (PL) measurements to understand how the Cs/FA ratio influences the formation of Cs_1-*x*_FA_*x*_PbI_3_ (*x* = 0, 0.25, 0.5, 0.75) PeNPLs. Our results reveal that Cs_1-*x*_FA_*x*_PbI_3_ with low values of *x* exhibit low nucleation and growth rates, leading to the formation of well-ordered PeNPLs. In contrast, FA-rich materials exhibit accelerated nucleation and growth kinetics, which induce overgrown and non-uniform PeNPLs. On the other hand, Cs-only PeNPLs demonstrate rapid degradation under ambient conditions, transitioning into the non-photoactive yellow *δ*-phase CsPbI_3_. In comparison, Cs/FA-alloyed PeNPLs exhibit improved stability in both colloidal solution (PL *t*_50_ = 210 min at 80 °C) and thin films (maintaining the α-phase over the 7-day test period) under ambient conditions. The resulting Cs/FA hybrid PeNPLs exhibit a degree of polarization (DOP) of 9.4%, significantly higher than the DOP of 5.1% observed for Cs-only PeNPLs. Our study on the nucleation and growth mechanisms of Cs/FA-based PeNPLs provides valuable insights into their formation process and identifies a promising synthetic pathway that enables their self-assembly into well-oriented superlattices through enhanced surface ligand interactions. This structural control contributes directly to the improved intrinsic and extrinsic stability, as well as efficient linearly polarized red-emissive PeNPLs.

## Results

### Synthesis of (Cs, FA)PbI_3_ alloy PeNPLs

**Fig. 1:**
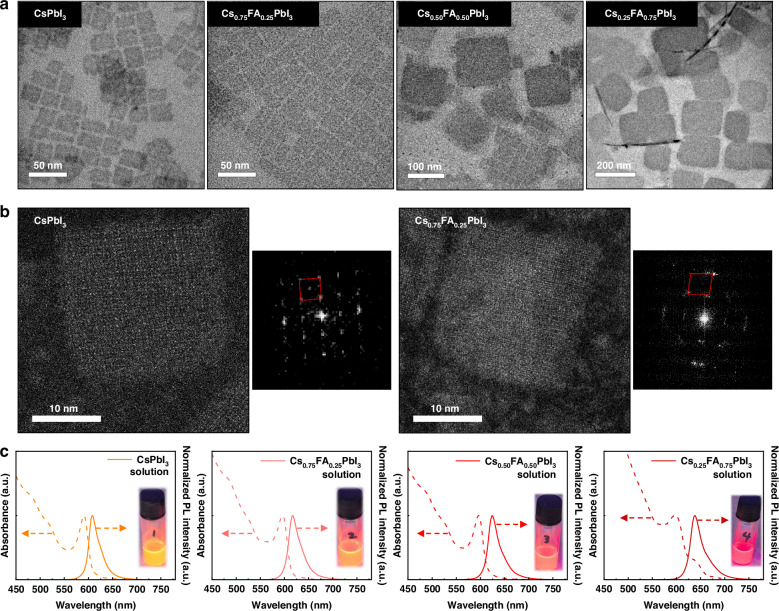
Characterization of Cs/FA-alloyed PeNPLs. **a** High resolution transmission electron microscopy (TEM) images of PeNPLs with different Cs/FA ratios. **b** Dark field scanning TEM images and fast Fourier transform of PeNPLs with 0% and 25% FA incorporation into the A-site. **c** Ultraviolet-visible absorption and photoluminescence spectra of colloidal PeNPL solutions. Inset are photographs of colloidal PeNPL solutions illuminated with a UV lamp (365 nm wavelength)

As a starting point, previous research has demonstrated that the structural stability of Cs_1-*x*_FA_*x*_PbI_3_ perovskites improves as the FA content increases^38,44,45^. However, these studies focused on bulk perovskites or weakly-confined PNCs, and Cs/FA alloying has not been explored as a strategy to enhance the stability of PeNPLs. Based on these findings, we hypothesized that the stability of well-ordered PeNPLs would improve with increasing FA content. As discussed in the introduction, PeNPLs require more meticulous synthetic control than isotropic, cube-shaped PNCs because anisotropic nanoplatelets are obtained under kinetically-driven conditions, whereas thermodynamically-governed conditions would favor isotropic nanocubes^9,46^. To investigate the mechanism of nucleation and growth, we synthesized Cs_1-*x*_FA_*x*_PbI_3_ (*x* = 0, 0.25, 0.50, 0.75) PeNPLs. The *x* value represents the ratio of Cs-oleate to FA-oleate injected into the PbI_2_-ligand solution during synthesis (see Methods), and therefore represents the nominal Cs/FA ratio. Transmission electron microscopy (TEM) images of each PeNPL sample are shown in Fig. 1a and Fig. S1 (Supplementary Information). The PeNPLs were dispersed in different solvents and drop-cast onto grids to obtain an edge-up (hexane) or face-down (octane) orientation. The evaporation rate, which is determined by the vapor pressure of the alkane solvent the PeNPLs are redispersed in after purification, affects the thermodynamic and kinetic processes that control the orientation of the nanoplatelets during film formation^12,16^. TEM analysis indicates that *x* = 0 and *x* = 0.25 PeNPLs exhibit a uniform two-dimensional platelet morphology with consistent dimensions, where the variation in the length of the PeNPLs was <13% of the median length. Figure 1b shows the dark-field scanning transmission electron microscopy (STEM) image and fast Fourier transform (FFT) patterns of the *x* = 0 and *x* = 0.25 PeNPLs. According to the FFT pattern, the *x* = 0 PeNPLs show a clear cubic crystal structure, whereas the *x* = 0.25 PeNPLs show a slightly tilted octahedral crystal structure due to structural distortion caused by the A-site alloying of Cs and FA cations^47–49^. While the thicknesses of these PeNPLs were the same (2.6 ± 0.4 nm), there was an improvement in the regularity of the shape of the PeNPLs with FA alloying. The median lengths of the PeNPLs were the same (26 ± 2 nm) for both compositions, while the widths increased from 21 ± 3 nm for *x* = 0 to 22 ± 2 nm for *x* = 0.25, leading to the aspect ratio (AR) reducing from 1.26 ± 0.19 to 1.09 ± 0.06 (Fig. S3, Supplementary Information). Previous studies have reported that PeNPLs with AR values closer to unity have improved stability^27^. We therefore expect that adding a small amount of FA is beneficial (see later for a detailed discussion). However, we found that further increasing the FA content led to overgrowth of the PeNPLs to >100 nm in size. For *x* = 0.50 PeNPLs, a broad size distribution was observed (Fig. S2, Supplementary Information), ranging from 20 nm to 160 nm, indicating the coexistence of small PeNPLs and overgrown PeNPLs. In contrast, *x* = 0.75 NPLs were predominantly composed of overgrown PeNPLs exceeding 100 nm.

A strong excitonic peak was observed in the UV-Vis absorption spectra of the colloidal PeNPLs (Fig. 1c). The exciton binding energies determined through Elliott model fitting were 212 meV (*x* = 0), 213 meV (*x* = 0.25), 165 meV (*x* = 0.50), and 153 meV (*x* = 0.75), respectively. For *x* = 0 and *x* = 0.25, the exciton binding energies are consistent with previous reports of CsPbI_3_ PeNPLs with 2.6 nm thickness^16^, which falls well below the exciton Bohr diameter (~12 nm)^50^. In the case of *x* = 0.75 PeNPLs, a relatively weak excitonic peak was observed, which can be attributed to nonuniform growth, with a heterogeneous size distribution caused by the overgrowth of PeNPLs. The PL emission peaks of each colloidal PeNPL solution were observed to be centred at 608, 616, 624, and 638 nm. These are all consistent with 3-monolayer thick PeNPLs, albeit with a slight red-shift in PL with increasing FA content in the A-site (Fig. 1c), consistent with an overall reduction in the degree of confinement.

Subsequently, X-ray diffraction (XRD) measurements were conducted for each PeNPL composition to investigate structural changes resulting from changing the FA content in the A-site of the perovskites. According to the XRD patterns (Fig. S5, Supplementary Information), increasing the FA cation content induces larger lattice parameters, which shift the diffraction peaks to smaller angles^38,40,51^. Furthermore, the XRD patterns of the *x* = 0 and *x* = 0.25 PeNPLs exhibit superlattice peaks, arising from diffraction from stacked PeNPLs arranged face down. Notably, the overall XRD peak intensities gradually decrease with increasing FA content, which can be attributed to the overgrowth of PeNPLs, leading to larger lateral dimensions and a heterogeneous size distribution, both of which contribute to more random stacking orientations.

### Growth kinetics of Cs/FAPbI_3_ alloy PeNPLs

**Fig. 2:**
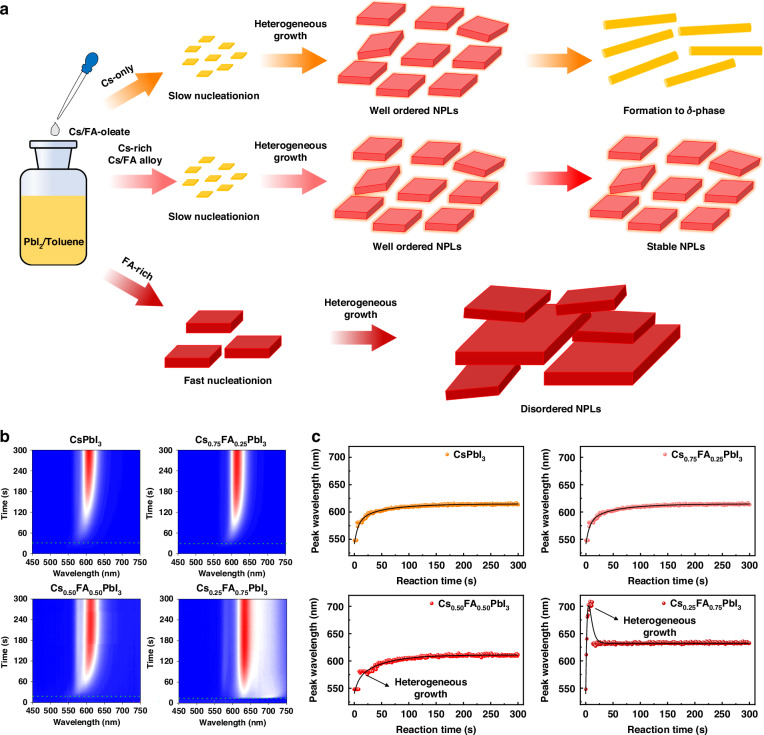
Nucleation and growth kinetics of PeNPLs. **a** Schematic illustration of the synthesis of PeNPLs with tunable Cs/FA ratios. **b** In-situ PL spectra during the formation of each PeNPL in colloidal solution. **c** Evolution of the PL peak wavelength over time after injecting the Cs/FA oleate solution into the reaction mixture. Data points were collected at 1 s time intervals

Forming a well-ordered PeNPL superlattice requires uniform PeNPLs. Our results in Fig. S5 (Supplementary Information) show that this is accomplished with low FA contents, but not when the fraction of FA in the A-site exceeds 0.5. This is due to increased disorder and less uniform sizes and shapes of the PeNPLs for high FA content. To explain this, we propose a mechanism that is illustrated in Fig. 2a. Specifically, we propose that *x* = 0 and *x* = 0.25 PeNPLs undergo slow nucleation with little structural distortion, leading to the formation of well-ordered PeNPLs through homogeneous growth. Conversely, we propose that faster nucleation takes place with increasing FA content, and that structural distortions are induced by FA cations, which have a different ionic radius than Cs^+^, disrupting the uniform growth of PeNPLs. This results in the formation of disordered PeNPLs with different sizes, which we describe as heterogeneous growth.

To experimentally test these hypotheses, we monitored the change in the PL spectra of the colloidal solution, illuminated with a 405 nm wavelength continuous wave (cw) laser, over time after injecting the Cs-oleate/FA-oleate precursor into the PbI_2_-ligand solution. We could assign the thickness of the PeNPLs obtained based on the PL peak wavelengths^16,18^. According to the in-situ PL spectra shown in Fig. 2b and Fig. S6 (Supplementary Information), we observed significant differences in the rates of nucleation and growth for each composition. For *x* = 0 PeNPLs, nucleation occurs approximately 30 s after the injection of Cs-oleate and starts with *n* = 2 nanoplatelets (~550 nm, depending on the Cs/FA composition), followed by growth to the *n* = 3 nanoplatelets (~610 nm) within 2 min (Fig. 2c). In the case of *x* = 0.25 PeNPLs, the growth kinetics are similar to those of *x* = 0 PeNPLs, with slightly faster nucleation and growth but still exhibiting overall homogeneous growth, in which PeNPLs with different thicknesses or perovskite nanocubes are not formed. In contrast, for compositions with *x* ≥ 0.5, different growth behavior was observed. For *x* = 0.50 PeNPLs, the PL peak positions over time reveal rapid formation of *n* = 2 ( ~ 550 nm) within the initial reaction period (~30 s), followed by slow growth to *n* = 3 nanoplatelets (~600 nm) (Fig. 2c). For FA-rich compositions, an intriguing phenomenon was observed during the growth of *x* = 0.75 PeNPLs. PL peaks centered at ~700 nm (likely corresponding to FAPbI_3_ PNCs) were detected within the first 10 s. Following this, the PL peak shifted to 631 nm within 8 s (Fig. 2c), which we attribute to FAPbI_3_ nanocrystals changing to (Cs, FA)PbI_3_ PeNPLs. However, the final PL of these PeNPLs is the most red-shifted out of all samples compared. For *x* = 0.50 and 0.75 PeNPLs, the PL spectra also had additional peaks alongside the main peak corresponding to the *n* = 3 PeNPLs (Fig. 2b and Fig. S6, Supplementary Information), indicating the co-existence of both low-*n* PeNPLs and perovskite nanocubes. These non-uniform species could hinder the formation of well-ordered PeNPL superlattices.

To further substantiate our understanding of the effects of FA on nanoplatelet growth, we investigated the nucleation and growth kinetics of FAPbI_3_ PeNPLs under equivalent synthesis conditions (Fig. S7, Supplementary Information). In-situ PL for these colloidal nanoplatelet solutions demonstrates nearly instantaneous nucleation upon injection, with almost all PeNPLs becoming *n* = 3 within the first 10 s (Fig. S7c, Supplementary Information). This behavior contrasts sharply with the slower nucleation and growth kinetics observed for CsPbI_3_ PeNPLs, providing compelling evidence to support the hypothesis that FA addition to CsPbI_3_ led to faster nucleation and growth. Among the compositions studied, CsPbI_3_ and Cs_0.75_FA_0.25_PbI_3_ PeNPLs emerged as the most promising candidates, providing optimal structural and optical properties, such as well-ordered PeNPLs with a narrow size distribution and the highest exciton binding energies, and these are the focus of further investigations, detailed next.

### Enhanced phase and optical stability of PeNPLs

**Fig. 3:**
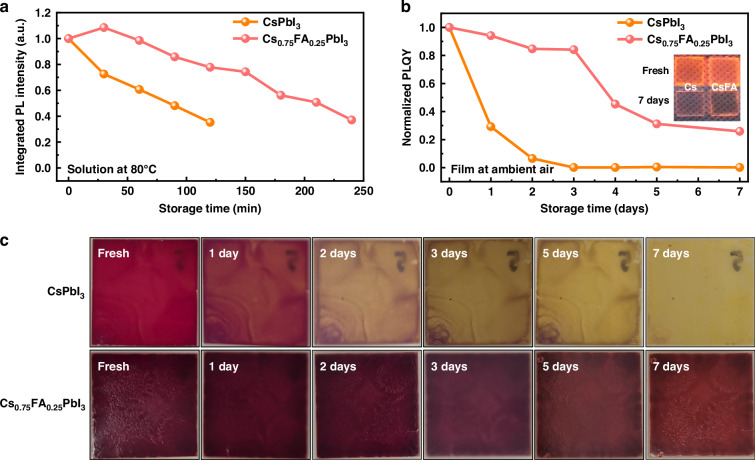
Optical and phase stability of PeNPLs. **a** Integrated PL intensity of colloidal PeNPL solutions as a function of time stored at 80 °C in ambient air. **b** Change in the normalized PLQY of PeNPL thin films. **c** Photographs of thick PeNPL films as function of time stored in ambient air

Based on the understanding of the synthesis and growth kinetics of PeNPLs, we investigate the effect of FA incorporation on the stability of PeNPLs. As a starting point, we heated a colloidal solution of CsPbI_3_ and Cs_0.75_FA_0.25_PbI_3_ to an elevated temperature (80 °C) and monitored the PL intensity over time (Fig. 3a and Fig. S8, Supplementary Information). As shown in Fig. 3a, the PL intensity of the *x* = 0 PeNPLs rapidly decreased, reaching 50% of the original PL intensity (*t*_50_) after 90 min. For the *x* = 0.25 PeNPLs, the *t*_50_ increased to 210 min. In general, surface ligands that dynamically bond to perovskite nanocrystals tend to detach more readily at elevated temperatures, leading to reduced colloidal and optical stability^52,53^. The decrease in PL intensity over time upon heating is attributed to the detachment of labile organic ligands from the surface of the PeNPLs and the degradation of the perovskite structure into the yellow *δ*-phase. To further investigate the role of FA incorporation in enhancing extrinsic stability, we conducted X-ray photoelectron spectroscopy (XPS) and ^1^H-NMR analysis on CsPbI_3_ and Cs_0.75_FA_0.25_PbI_3_ PeNPL dispersions before and after 7 days of ambient aging. The N/Pb atomic ratio, which reflects the amount of surface ligands, showed a significant decrease in CsPbI_3_ PeNPLs, indicating notable ligand detachment (Fig. S9 and Table S1Supplementary Information). In contrast, the Cs_0.75_FA_0.25_PbI_3_ PeNPLs retained a higher N/Pb ratio, suggesting stronger ligand binding and improved surface passivation. ^1^H-NMR analysis further confirmed a more significant loss of surface ligands in CsPbI_3_ PeNPLs after aging, while the Cs_0.75_FA_0.25_PbI_3_ PeNPLs exhibited minimal ligand detachment, indicating improved ligand binding (Fig. S11, Supplementary Information). These results therefore suggest that FA addition not only reduces thermally induced ligand detachment but also improves the overall extrinsic stability of the PeNPLs by mitigating surface degradation pathways under environmental conditions.

To measure the stability of the PeNPLs when assembled together to form films, we prepared thin films by spin coating onto glass substrates, as well as thick films by drop casting the colloidal solution onto glass substrates. We monitored the stability of these films in air under ambient conditions (40% relative humidity, 20 °C, storage in the dark). For the thin films, we monitored the PLQY over time, and found that the PLQY of the *x* = 0 PeNPLs reduced to 10% of the original value within 2 days, whereas the *x* = 0.25 PeNPL film maintained 84% of the original PLQY over the course of 3 days, before reducing to 30% of the initial PLQY after 7 days (Fig. 3b). To monitor the phase stability, we measured changes in the diffraction pattern of the thick films stored in air under the same conditions (Fig. 3c and Fig. S12, Supplementary Information). For the *x* = 0 PeNPLs, the yellow *δ*-phase started to appear after just 1 day, after which the film rapidly became the *δ*-phase after 3 days. The *x* = 0.25 PeNPL films were more stable, mostly maintaining the α-phase over the 7-day testing period, with only traces of *δ*-phase appearing from day 3 onwards.

### Effect of FA on strength of ligand binding to perovskite surface

**Fig. 4:**
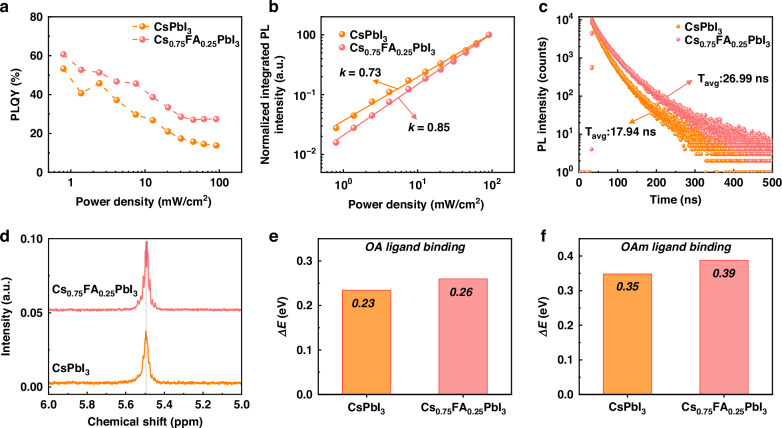
Optical properties and surface chemistry of PeNPLs. Power density-dependent **a** PLQY and **b** integrated PL peak intensity measurements, along with **c** time-resolved PL decay curves of colloidal PeNPL solutions. **d** ^1^H-NMR spectra of colloidal PeNPLs dispersed in deuterated toluene. DFT-calculated binding energy of **e** oleic acid and **f** oleylamine on the surface of each PeNPL surface

To understand the influence of FA alloying on the optoelectronic properties of PeNPLs, we conducted excitation power dependent PLQY and time-resolved photoluminescence (TRPL) measurements (Fig. 4a–c and Fig. S13, Supplementary Information). The PLQY measurements of each PeNPL dispersion revealed that the *x* = 0.25 PeNPLs exhibited higher values, reaching a maximum of 61% (median: 59%), whereas the *x* = 0 PeNPLs showed a maximum of 49% (median: 48%) (Fig. S13, Supplementary Information). As shown in Fig. 4a, at all excitation power densities, the PLQY values of *x* = 0.25 PeNPLs were higher than those of *x* = 0 PeNPLs. The PLQY decreased with increasing excitation power density in both cases (refer to Fig. S14, Supplementary Information for PL spectra). Given the high exciton binding energies (refer to earlier section on Elliott model fitting), this decrease in PLQY with increasing excitation power density is due to bimolecular exciton-exciton annihilation. Fitting a power law function to the PL intensity as a function of excitation power density yields exponents close to unity (Fig. 4b). This is consistent with excitonic behavior, whereas a sublinear dependence would typically indicate the involvement of non-radiative pathways such as trap-assisted recombination or exciton-exciton annihilation^16^. We fit the PL decay curves with a phenomenological multi-exponential decay function (Fig. 4c). The weighted average of the time constants fit to quantitatively describe the PL decays (τ_avg_) increased from 17.94 ns (*x* = 0) to 26.99 ns (*x* = 0.25). There was therefore a consistent increase in PLQY and TRPL τ_avg_ from *x* = 0 to *x* = 0.25, suggesting that the enhancement in τ_avg_ is due to fewer non-radiative recombination processes with a small amount of FA incorporation to the PeNPLs.

The surface ligands (here oleic acid OA, and oleylamine OAm) play a key role in enhancing the optoelectronic properties and stability of PeNPLs through the suppression of surface defects. These ligands also play a critical role on long-term stability by reducing the exposure of the surface of the perovskite to moisture and oxygen, which can cause PeNPL degradation^52–54^. We analyzed the ligand density on the PeNPLs through solution phase ^1^H-NMR spectroscopy (Fig. 4d; full spectra in Fig. S15, Supplementary Information). We obtained the NMR spectrum from 3 mg of PeNPLs and 0.5 mg of ferrocene dispersed in 1 mL of deuterated toluene (d-toluene) solution. As shown in Fig. 4d, the integration of the NMR peaks was performed with respect to the ligand peak at a chemical shift of around 5.5 ppm, which corresponds to the vinyl peaks (C=C) from both the oleic acid and oleylamine ligands^55^. From this, we found that the *x* = 0.25 PeNPLs had about 1.2 times higher ligand density than the *x* = 0 PeNPLs. In addition, a slight shift of the ligand peak from 5.49 ppm to 5.48 ppm was observed in the *x* = 0.25 PeNPLs compared to *x* = 0 PeNPLs. This is consistent with the formation of hydrogen bonding between the FA cation and the ligands, leading to the increase in ligand density observed for these PeNPLs, and is consistent with observations previously reported for perovskite NCs with FA cations^38,56^.

To gain a deeper understanding of the binding properties of each ligand (OA, OAm) on the PeNPL surface, we performed density functional theory (DFT) calculations to determine the binding energies of these ligands with *x* = 0 and *x* = 0.25 perovskites. The DFT-calculated binding energies for each perovskite surface and ligand are presented in Fig. 4e, f, and the slab model of the perovskite structure is shown in Fig. S16, Supplementary Information. The results indicate that FA cations on the PeNPL surface are involved in hydrogen bonding with the functional groups of the ligands, as evidenced by the increased binding energies of OA^–^ and OAm^+^ ligands on the *x* = 0.25 PeNPLs compared to the *x* = 0 PeNPLs (Fig. 4e, f). The enhanced ligand density and stronger surface binding energy between the ligands and PeNPLs account for the reduced non-radiative recombination observed in Cs/FA alloyed PeNPLs. These findings support the hypothesis that *x* = 0.25 PeNPLs enable stronger ligand passivation on the PeNPLs surface as well as structural thermodynamic stability, and suggest a pathway to achieve highly emissive, long-term stable PeNPLs.

### Linearly-polarized photoluminescence from PeNPL films

**Fig. 5:**
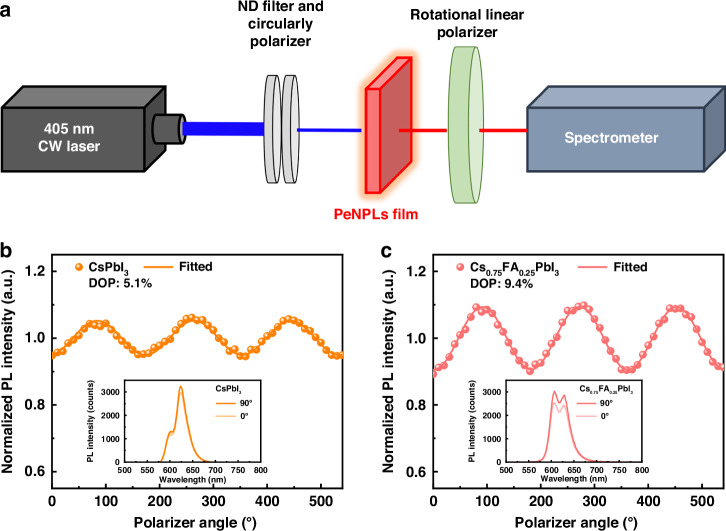
Linearly polarized emission of PeNPL films. **a** Experimental setup for measuring the degree of linear polarization (DOP) in PL. **b** Polarization dependence of the normalized PL intensity of **b** CsPbI_3_ and **c** Cs_0.75_FA_0.25_PbI_3_ PeNPL film. Inset are the PL spectra as a function of polarizer angle

As outlined in the introduction, light emitters with strongly confined and anisotropic structures, particularly two-dimensional PeNPLs, can exhibit linearly polarized emission, making them highly promising for numerous optical applications. Based on the successful synthesis and control over the uniformity, stability, and optoelectronic properties of the PeNPLs obtained in this work, we measured the degree of polarization (DOP) from ensembles of these PeNPLs in films made by drop-casting. The experimental setup for DOP measurements is shown in Fig. 5a. The PeNPL film was fabricated on a glass substrate by drop-casting a colloidal PeNPL solution that had hexane as the solvent. The rapid evaporation enabled PeNPLs to be kinetically trapped in the edge-up orientation. As shown in Fig. 5b, c, the PeNPLs exhibited a maximum DOP of 5.1%, with a median value of 4.3% across 6 samples for *x* = 0 PeNPLs. This increased to 9.4% (median value: 9.0% across 6 samples) for *x* = 0.25 PeNPLs. Histograms showing the distribution in DOP values measured are shown in Figs. S17 and S18, Supplementary Information.

The enhanced DOP for *x* = 0.25 can be attributed to three factors. Firstly, the improvement in surface ligand density, as discussed earlier, likely results in denser PeNPL arrangement during superlattice formation, which enhances linear polarization properties. This is further supported by grazing-incidence wide-angle X-ray scattering (GIWAXS) analysis (Fig. S19, Supplementary Information), which reveals that the *x* = 0.25 PeNPLs exhibit enhanced vertical orientation, indicative of more densely packed and directionally aligned superlattices. Secondly, the reduction in non-radiative recombination losses, enabled by effective defect passivation through Cs/FA alloying, contributes to a higher fraction of radiative exciton recombination. Since exciton recombination is inherently influenced by the anisotropic crystal structure of PeNPLs, reducing trap-assisted recombination enables the intrinsic polarization of excitons to emerge more prominently, leading to improved linear polarization properties. Thirdly, as shown inset in Fig. 5b, c, the *x* = 0.25 PeNPLs experienced reduced agglomeration than the CsPbI_3_ PeNPLs when assembled to form thin films, such that there was a larger contribution in their emission from the more strongly-confined *n* = 3 PeNPLs. This would lead to enhanced exciton fine structure splitting, which is needed to achieve a higher DOP. Overall, these enhanced linear polarization properties of PeNPLs highlight their potential as efficient and stable red emitters for next-generation display technologies.

## Discussion

In this work, we demonstrated that A-site hybridization through Cs/FA alloying significantly enhances the phase stability and optical properties of red-emitting I-based PeNPLs. By investigating the nucleation and growth kinetics, we show that low FA alloying results in slower nucleation and homogeneous growth, forming well-ordered structures, while FA-rich PeNPLs exhibit rapid nucleation and heterogeneous growth, leading to structural disorder. The optimized Cs/FA PeNPLs showed superior phase stability in thin films under ambient conditions, maintaining their photoactive phase for over seven days, compared to the rapid degradation observed in Cs-only PeNPLs. Additionally, Cs/FA alloying enhanced the interaction between surface ligands and the PeNPLs surface through hydrogen bonding, resulting in increased ligand density and improved radiative recombination. As a result, the Cs/FA alloy PeNPLs exhibited a 1.8-fold improvement in their emission of linearly polarized light, with a degree of polarization of 9.4%, compared to 5.1% for Cs-only PeNPLs. This enhancement suggests that Cs/FA alloy PeNPLs could serve as promising materials for applications requiring polarized light emission, such as optical communication, quantum emitters, and optoelectronic devices.

## Materials and Methods

### Materials

Cesium acetate (99.9%), formamidinium acetate (99%), oleylamine (OAm; 70% tech.), oleic acid (OA; 90% tech.), octane (anhydrous ≥99%), ferrocene (98%), toluene (anhydrous 99.8%), and hexane (anhydrous 95%) from Sigma Aldrich. Lead iodide (PbI_2_; 99.999%) was purchased from Alfa Aesar.

### Synthesis of Cs_1-*x*_FA_*x*_PbI_3_ PeNPLs

Cesium oleate and formamidinium oleate precursors were prepared by dissolving 38.9 mg of cesium acetate or 21.5 mg of formamidinium acetate in 4 mL of oleic acid (0.05 mol L^−1^) and stirring at 80 °C for 1 h. Lead iodide (PbI_2_) precursor was prepared by dissolving 46.1 mg of PbI_2_ powder in 10 ml toluene (0.01 mol L^−1^) with 100 µL oleic acid and 100 µl oleylamine and stirring at 120 °C for 4 h. For the synthesis of CsPbI_3_ PeNPLs, 8 mL of the PbI_2_ precursor solution was transferred into a hotplate at 40 °C. Subsequently, 240 µL of Cs-oleate solution was swiftly injected into the heated solution and allowed to react for 2 min. After reaction, we centrifuged at 12 000 rpm for 10 min, before discarding the supernatant, and redispersing the precipitate in hexane or octane. For the synthesis of Cs/FA alloy PeNPLs, Cs/FA-oleate is prepared by mixing the prepared Cs-oleate and FA-oleate according to their volume ratio (3:1, 2:2, 1:3), and then injected into PbI_2_ solution to synthesize Cs/FA alloyed PeNPLs. The purification was the same as for the synthesis of CsPbI_3_ PeNPLs.

### Characterization

Power-dependent PLQY was measured following the procedure by de Mello et al.^57^. Samples were placed in an integrating sphere and excited with a 405 nm wavelength CW laser. Film-state PLQY measurement was conducted with a commercial PLQY setup (Quantaurus-QY Absolute PL quantum yield spectrometer HAMAMATSU), and samples were excitation at 405 nm with a xenon lamp. The intensity of the laser was measured with Si photodiode, and the signal was collected with QEPro spectrometer. Steady-state PL measurements were carried out using a 405 nm CW (37.3 mW cm^−2^) intensity of the laser, and the signal was collected with QE Pro (Ocean Optics). And time-resolved PL decay measurements were carried out using a He–Cd laser operating at a wavelength of 375 nm with the FluoTime 30 system. XRD patterns were measured using a Bruker D8. ^1^H NMR spectra were measured with a 400 MHz NMR spectrometer (Bruker AVIII 400 Nanobay). UV-Vis absorption spectra were measured with a Shimadzu UV-2600 UV-Vis spectrometer. TEM samples were prepared by dropping a PeNPLs dispersion in hexane and octane on a carbon grid. TEM was measured with JEM-2100F model (JEOL). STEM was measured under cryogenic conditions (100 K) with JEM–ARM300F model (JEOL). Grazing incidence wide-angle X-ray scattering (GIWAXS) measurements were performed from Pohang Accelerator Laboratory 9 A U-SAXS Beamline with an incident angle of 0.12°. XPS spectra were measured using a X-ray photoelectron spectrometer NEXSA-G2 (ThermoFisher)

### Density functional theory

The DFT calculations employed a 2 × 2 × 1 slab model as the substrate for surface adsorption studies. The DFT calculations were conducted using density functional theory within the generalized gradient approximation with the Perdew−Burke−Ernzerhof (PBE) functional^58^ for the exchange−correlation energy, as implemented in the Vienna Ab Initio Simulation Package (VASP) code^59^. The plane-wave kinetic energy cutoff of 600 eV for the plane-wave expansion and Monkhorst-Pack **k**-point mesh resolution of 13 × 13 × 1 in reciprocal space for all of the structures in the Brillouin zonewere chosen to ensure an energy convergence and residual forces within 1 meV/atom and 1 meV/Å, respectively. The electron localization function (ELF) was calculated and plotted to explore the bonding mechanism of surface adsorption. The adsorption behavior of lead (Pb) atoms on the perovskite (001) surface was investigated using a 2 × 2 × 1 slab model, with systematic studies focusing on two distinct adsorption configurations: oxygen atoms from oleic acid interacting with Pb sites and nitrogen atoms from oleylamine binding to the Pb surface. The substrate geometry was optimized to preserve periodic boundary conditions while minimizing spurious interactions from periodic images. Density functional theory (DFT) calculations within the generalized gradient approximation (GGA-PBE)^58^ were performed using the Vienna Ab Initio Simulation Package (VASP)^59^, employing a 600 eV plane-wave cutoff and 13 × 13 × 1 Monkhorst-Pack k-mesh to ensure energy convergence (<1 meV/atom) and residual forces (<1 meV/Å). Electron localization function (ELF) analysis was conducted to characterize interfacial bonding characteristics.

## Supplementary information

Supplementary Information

## Data Availability

Raw data for the main text and supplementary information are available from the Oxford Research Archive repository, with the link 10.5287/ora-wvrdrgobg.
