# Supplementary material for: Enhanced stability and linearly polarized emission from CsPbI3 perovskite nanoplatelets through A-site cation engineering
Source: Light Sci Appl. 2026 Jan 2;15:22. doi: 10.1038/s41377-025-02135-y (PMC12757592; doi:10.1038/s41377-025-02135-y)
Supplement: Supplementary file 1 — Supplementary Information [file 41377_2025_2135_MOESM1_ESM.docx]

**Supplementary Information for**

**Enhanced Stability and Linearly Polarized Emission from CsPbI_3_ Perovskite Nanoplatelets through A-site Cation Engineering**

Woo Hyeon Jeong^1,2^, Junzhi Ye^2^*, Jongbeom Kim^3^, Rui Xu^4^, Xinyu Shen^1,5^, Chia-Yu Chang^2^, Eilidh L. Quinn^2^, Hyungju Ahn^6^, Myoung Hoon Song^3^, Peter Nellist^7^, Henry J. Snaith^5^, Yunwei Zhang^4^, Bo Ram Lee^1^* and Robert L. Z. Hoye^2^*

^1^ School of Advanced Materials Science and Engineering, Sungkyunkwan University, Suwon, 16419, Republic of Korea

^2^ Inorganic Chemistry Laboratory, University of Oxford, Oxford, OX1 3QR, United Kingdom

^3^ Department of Materials Science and Engineering, Ulsan National Institute of Science and Technology (UNIST), Ulsan, 44919 Republic of Korea

^4^ School of Physics, Sun Yat-sen University, Guangzhou, 510275 China

^5^ Clarendon Laboratory, Department of Physics, University of Oxford, Oxford, OX1 3PU United Kingdom

^6^ Pohang Accelerator Laboratory (PAL) Pohang, Gyeongbuk 37673, Republic of Korea

^7^ Department of Materials, University of Oxford, Oxford OX1 3PH United Kingdom

*Correspondence: junzhi.ye@chem.ox.ac.uk (J.Y.), brlee@skku.edu (B.R.L.), robert.hoye@chem.ox.ac.uk (R.L.Z.H.)

**
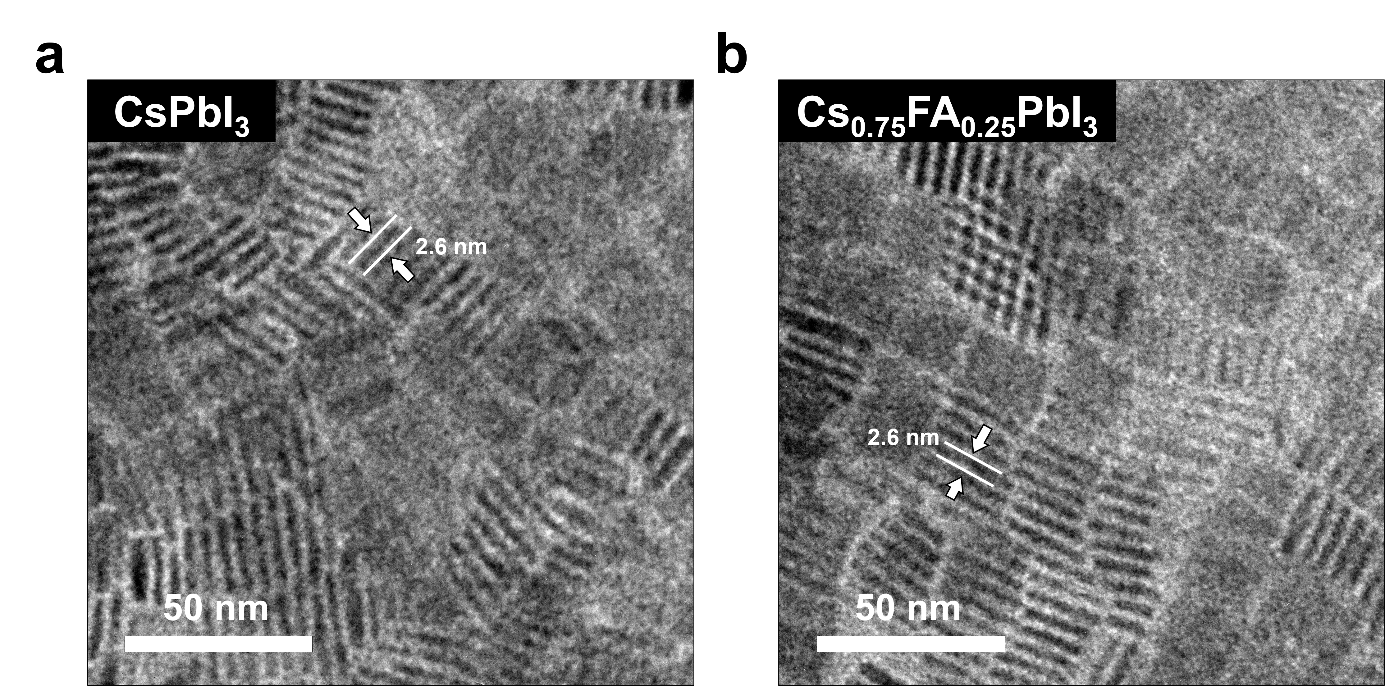
**

**Figure S1.** Transmission electron microscopy (TEM) image of **a** CsPbI_3_ and **b** Cs_0.75_FA_0.25_PbI_3_ PeNPLs. These nanoplatelets were dispersed in a hexane solvent, and drop cast onto a Cu TEM grid.


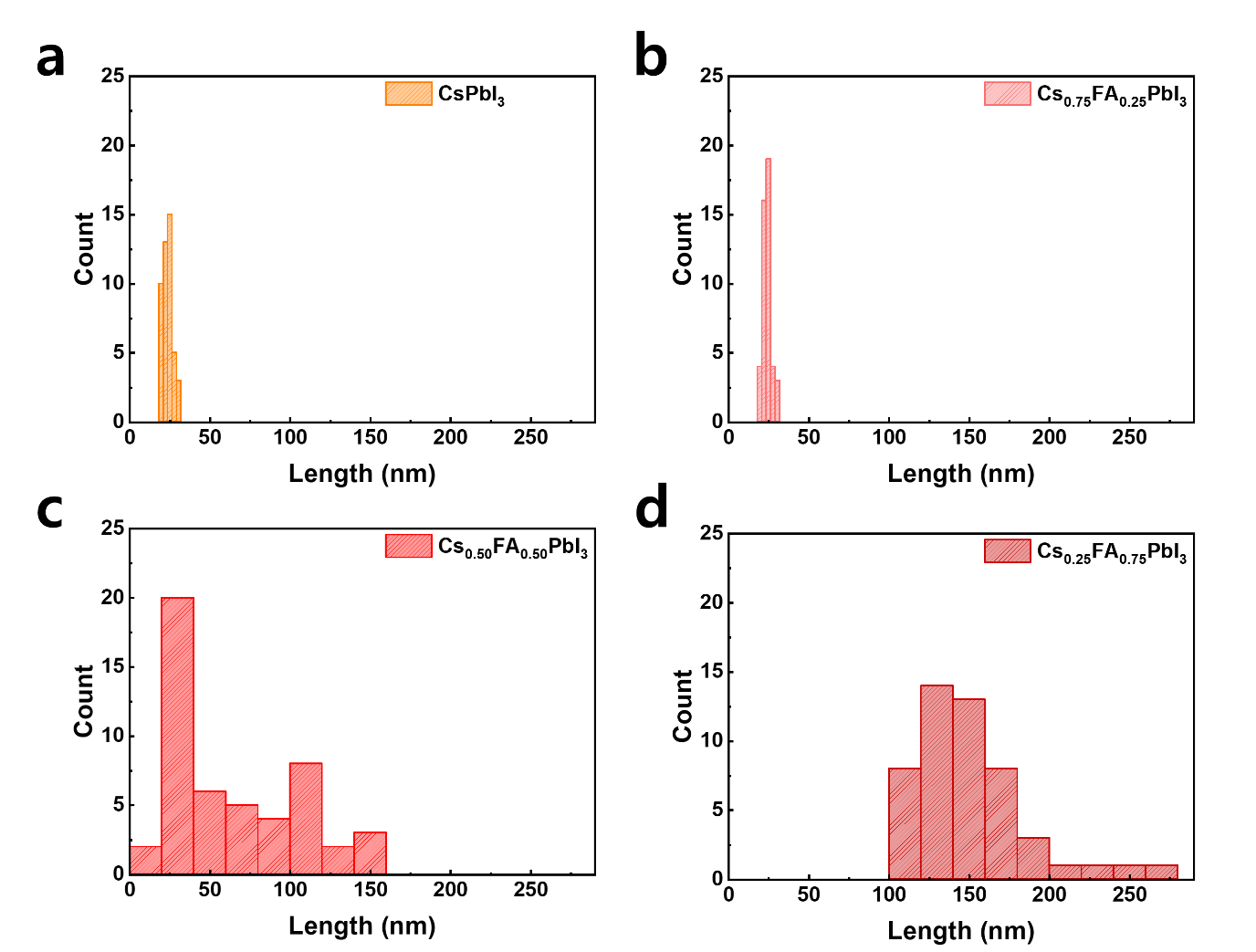


**Figure S2.** Size distribution histogram of **a** CsPbI_3_, **b** Cs_0.75_FA_0.25_PbI_3_, **c** Cs_0.50_FA_0.50_PbI_3_ and **d** Cs_0.25_FA_0.75_PbI_3_, determined from TEM measurements from Fig. 1a in the main text.


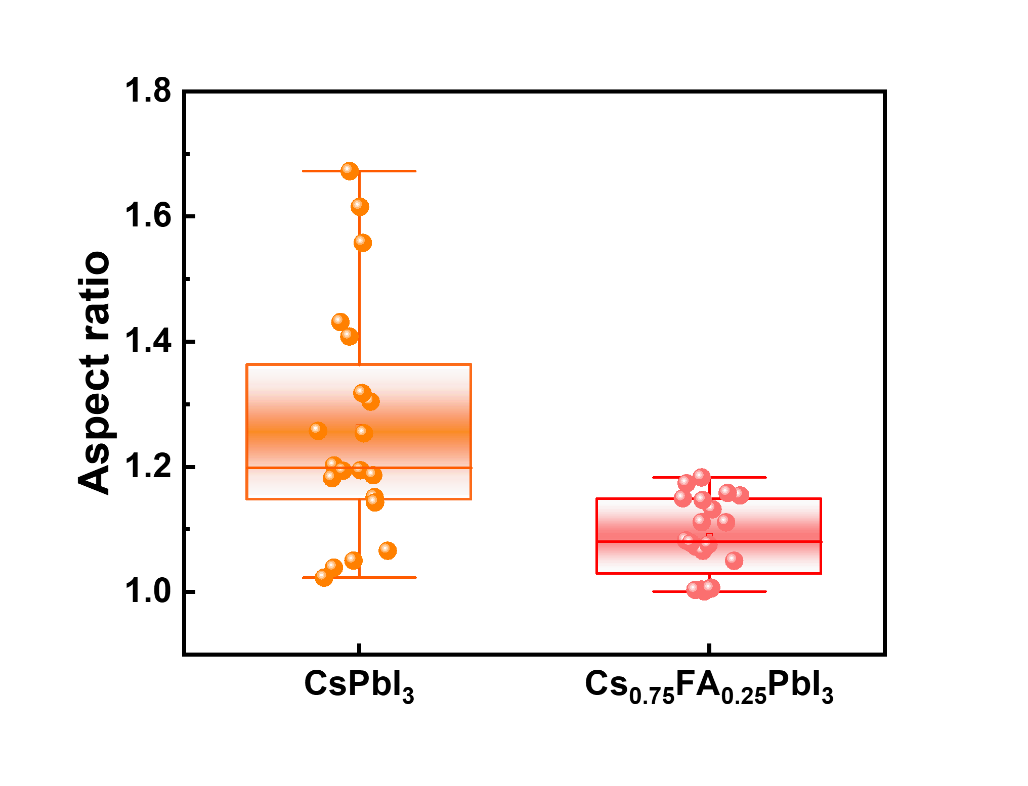


**Figure S3.** Distribution in the aspect ratio of PeNPLs. Data was collected from 20 individually-identified PeNPLs from Fig. S1.

**
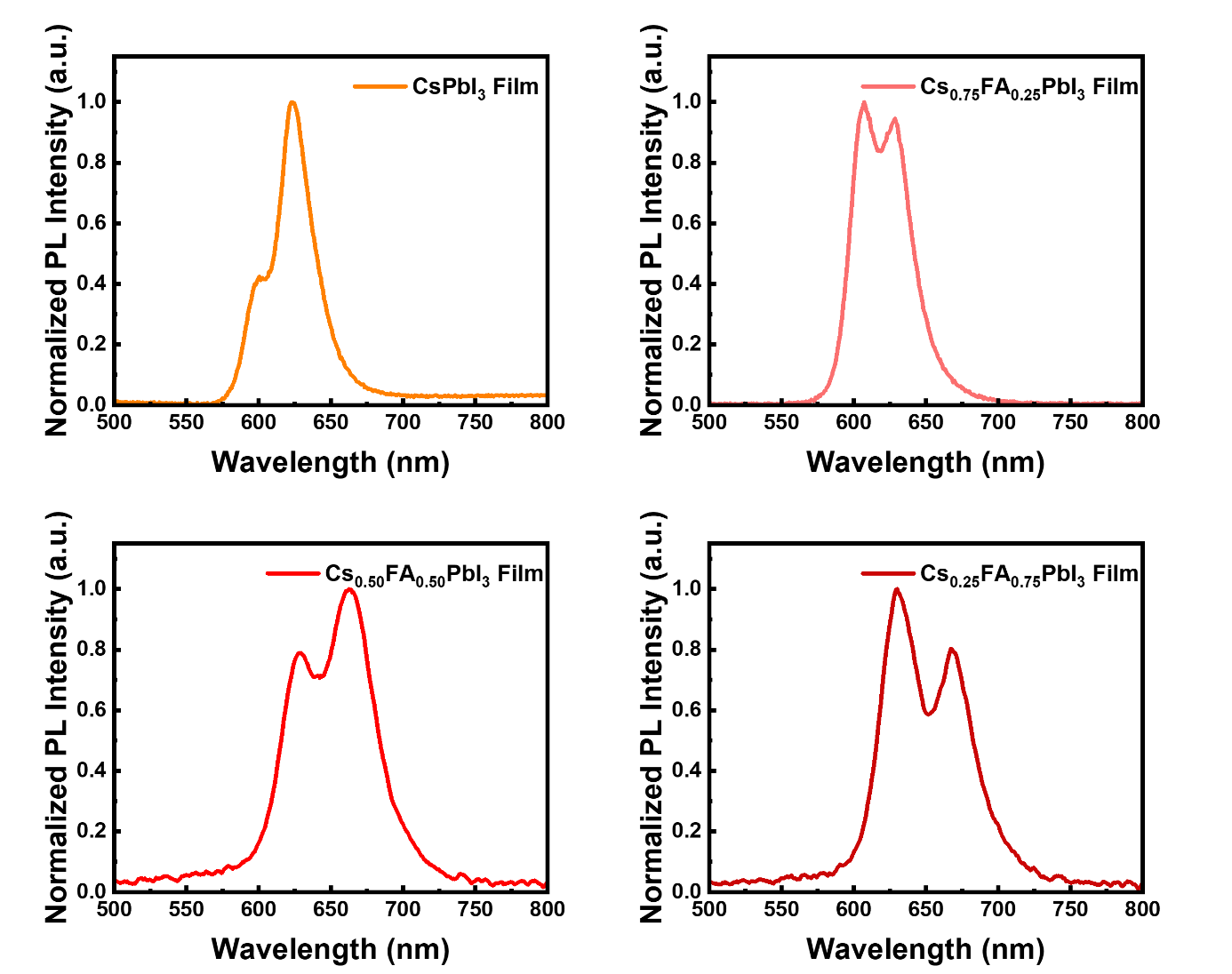
**

**Figure S4.** Photoluminescence spectra of (Cs,FA)PbI_3_ PeNPLs drop-cast onto glass substrates. Samples were measured in an N_2_-filled glovebox, excited with a 405 nm wavelength CW laser at 37.3 mW cm^-2^ power density.

**
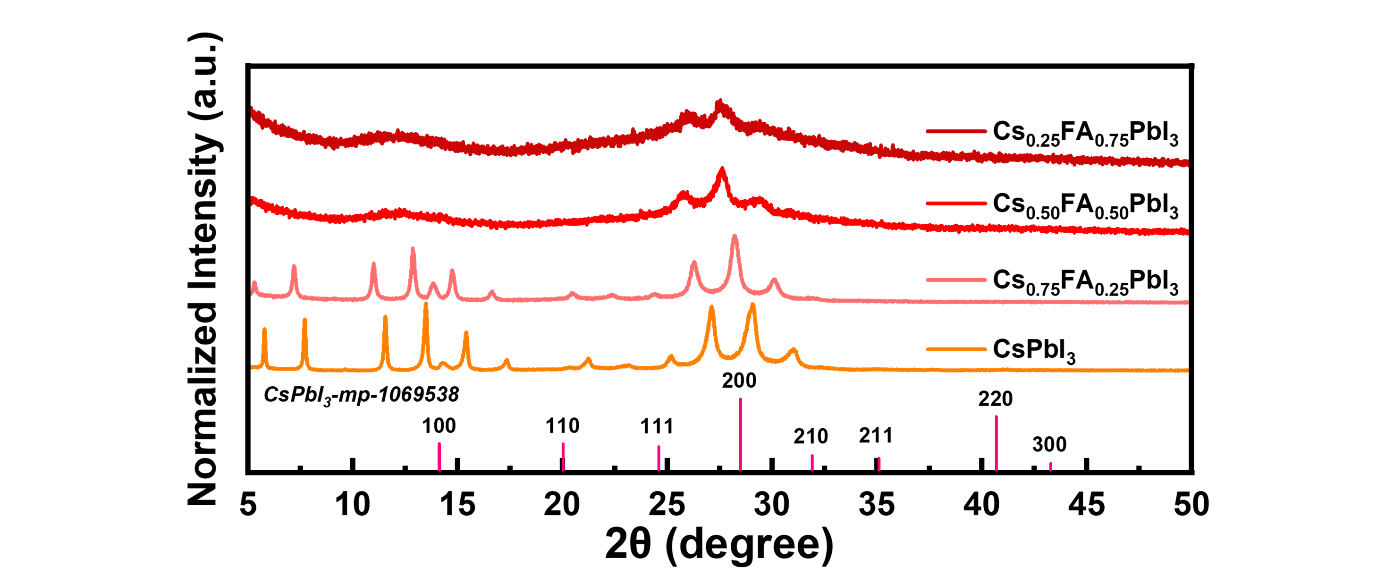
**

**Figure S5.** X-ray diffraction patterns of PeNPLs compared with the reference pattern for α-CsPbI_3_ (mp-1069538)^1^.

**
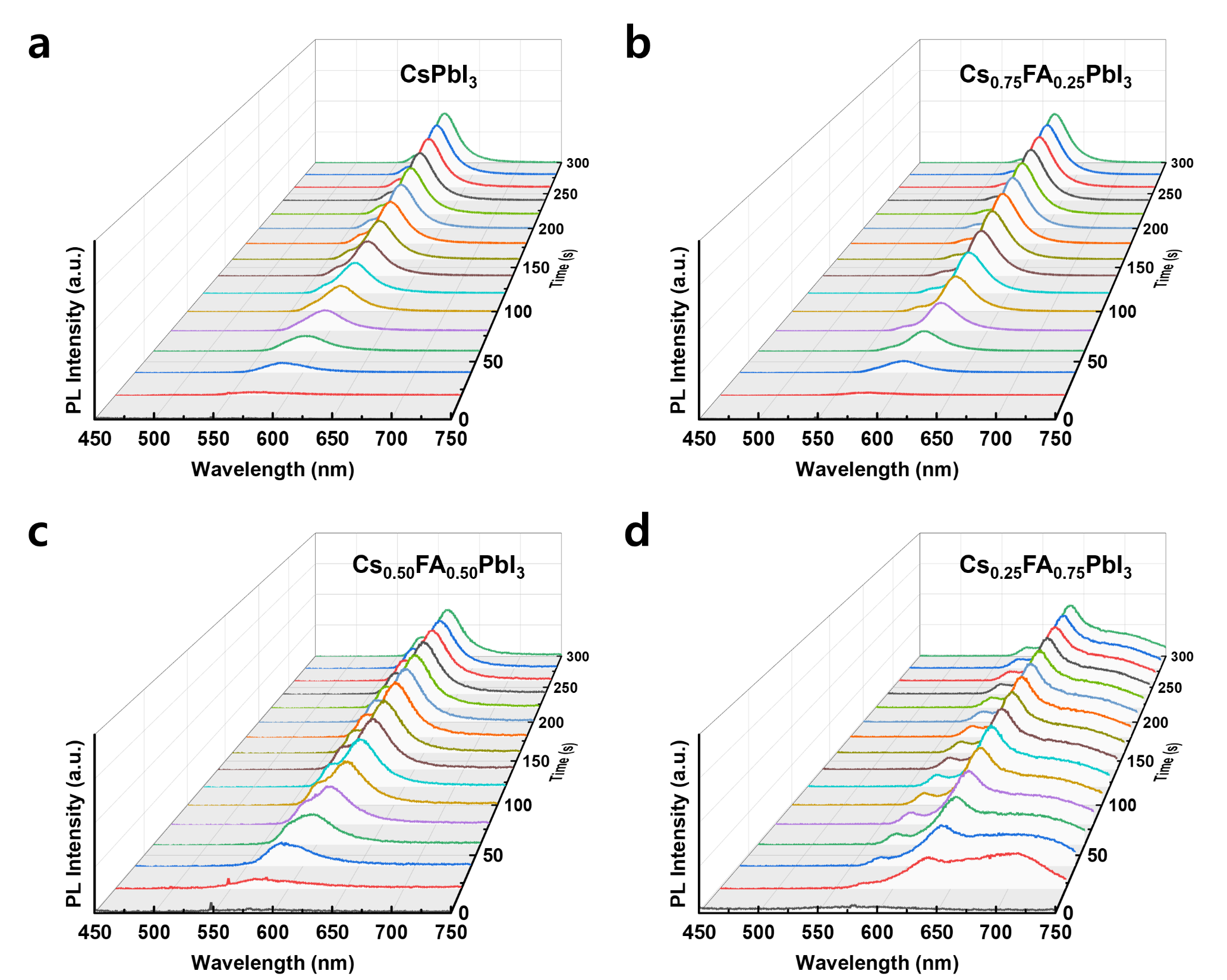
**

**Figure S6.** *In-situ* PL spectra during the formation of each PeNPL following the injection of the Cs-oleate/FA-oleate into the PbI_2_-ligand solution. These spectra are displayed in Fig. 2b in the main text. Spectra obtained at 20 s time intervals. Samples were measured in ambient air during synthesis (see Methods in the main text), excited with a 405 nm wavelength CW laser at 37.3 mW cm^-2^ power density.

**
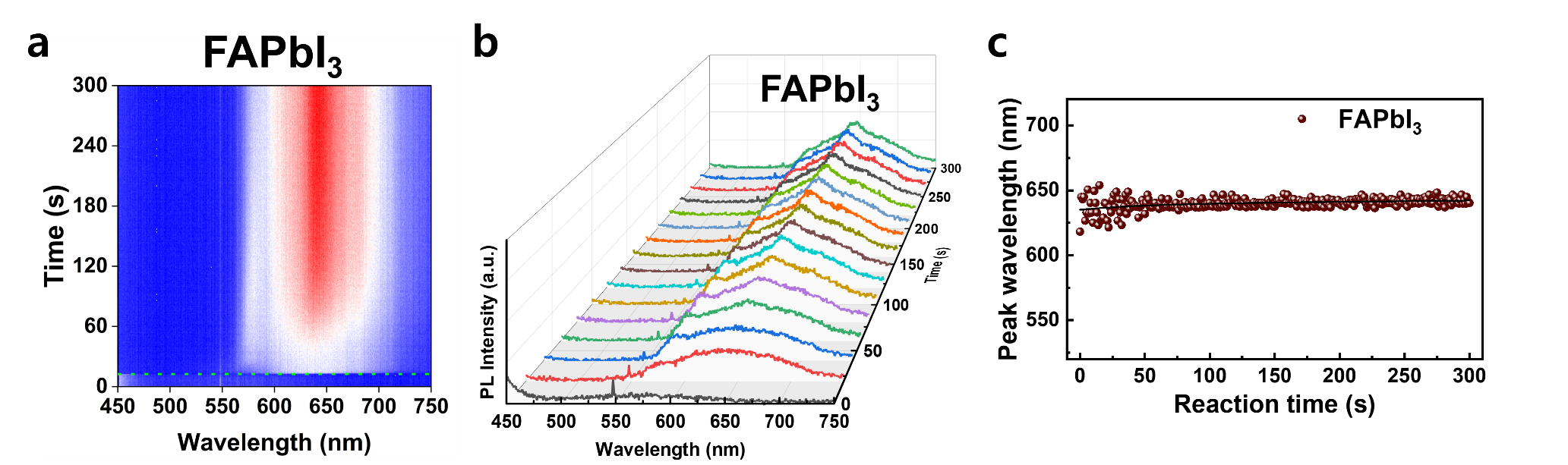
**

**Figure S7.** **a**, **b** *In-situ* PL spectra obtained during the formation of FAPbI_3_ PeNPLs. Spectra were collected at 20 s intervals from the time the FA-oleate was injected into the PbI_2_-ligand solution. **c** Detailed kinetics of the photoluminescence peak wavelength evolution over time after injecting the FA oleate solution into the reaction mixture. Data points were collected at 1 s time intervals. Samples were measured in an in ambient air during synthesis (see Methods in the main text), excited with a 405 nm wavelength CW laser at 37.3 mW cm^-2^ power density.

**
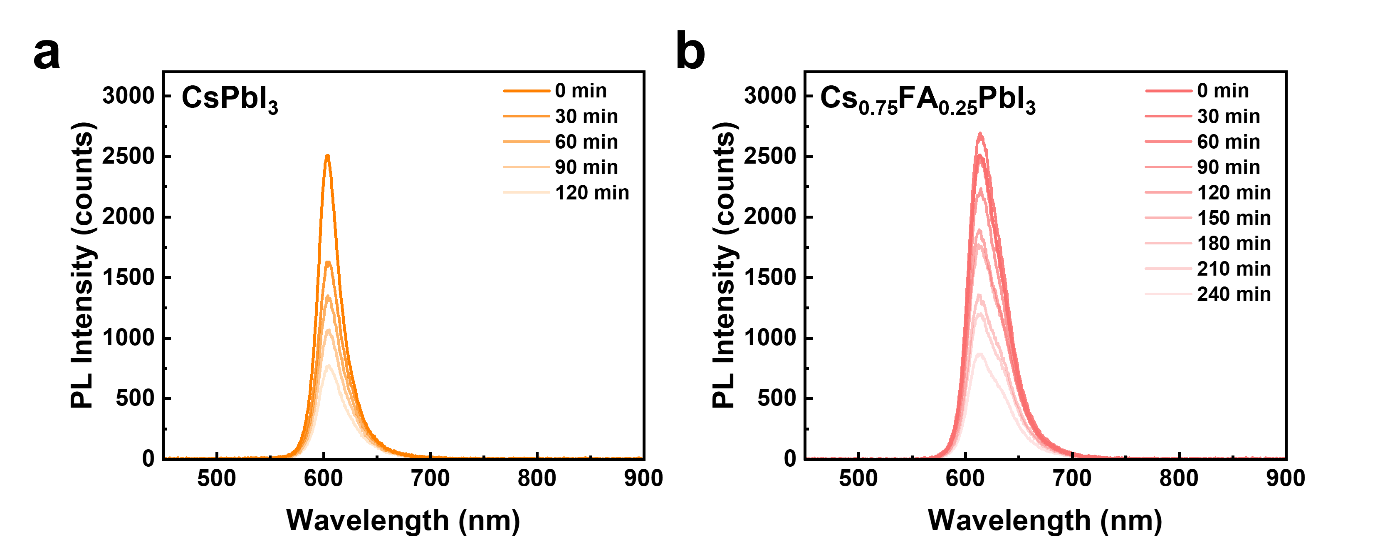
**

**Figure S8**. PL spectra of colloidal PeNPL solutions of **a** CsPbI_3_ and **b** Cs_0.75_FA_0.25_PbI_3_ heated to 80 °C as a function of time 0 to 240 min. Samples were measured at 80 ℃ in ambient air, excited with a 405 nm wavelength CW laser at 37.3 mW cm^-2^ power density.


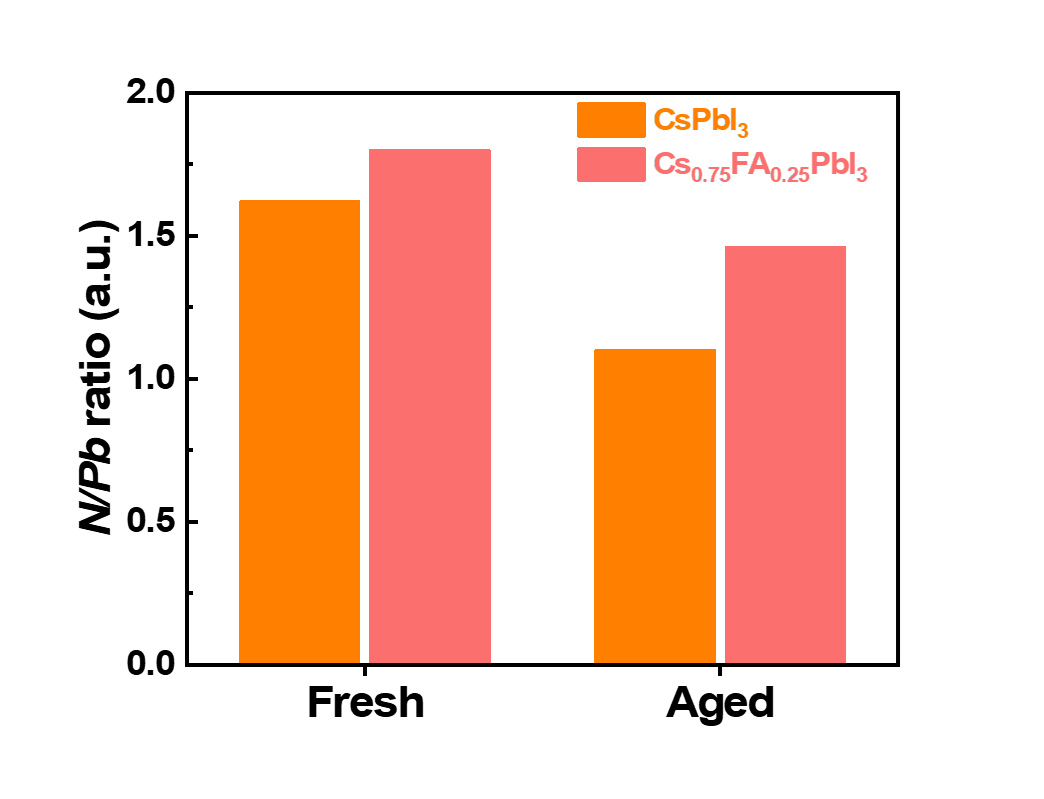


**Figure S9**. N/Pb ratios from XPS measurements of in CsPbI_3_ and Cs_0.75_FA_0.25_PbI_3_ PeNPLs before and after 7 days of ambient aging.


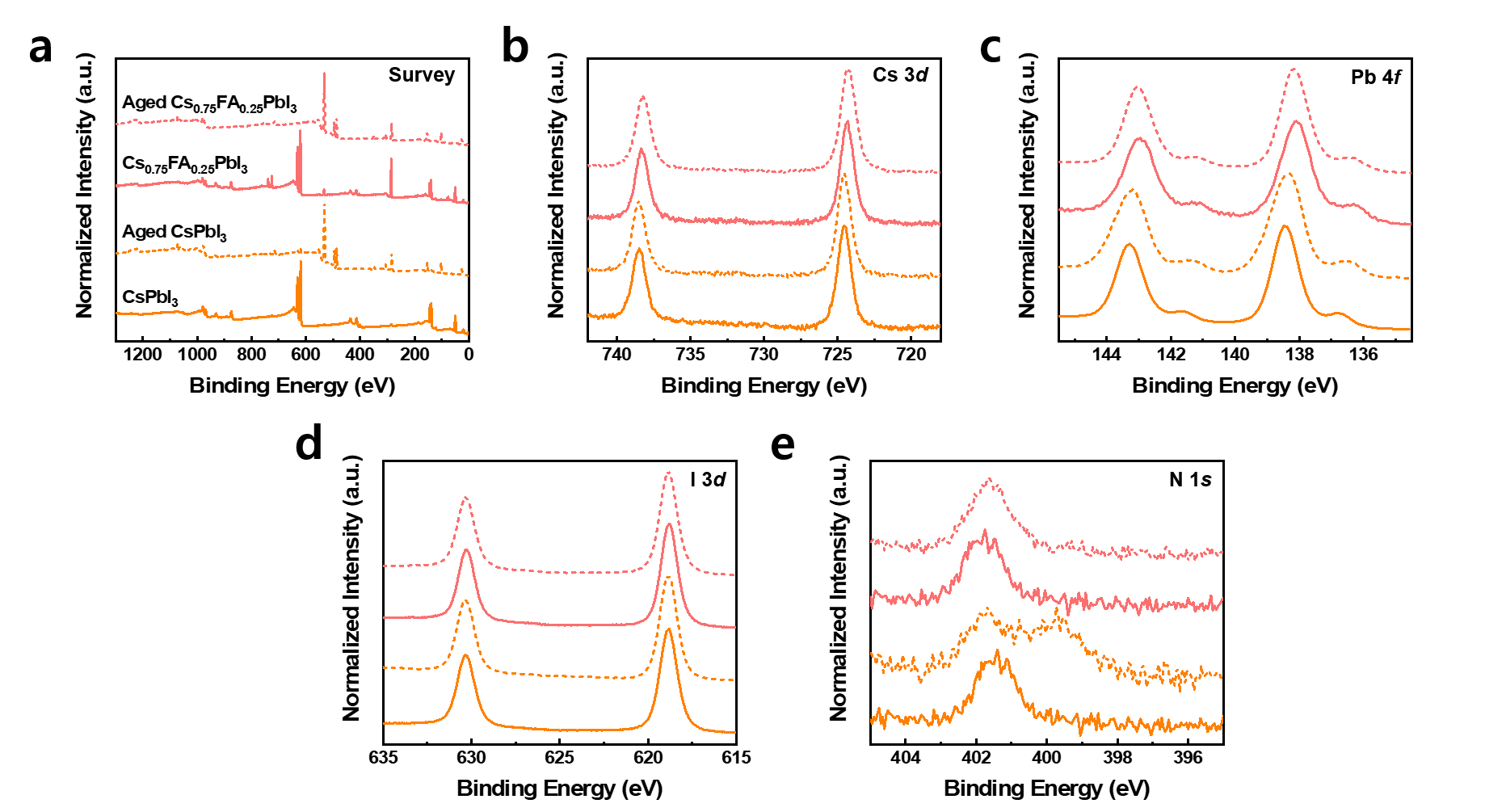


**Figure S10**. **a** Survey XPS spectra, **b** Cs 3d, **c** Pb 4f, **d** I 3d and **e** N 1s core level spectra of CsPbI_3_ and Cs_0.75_FA_0.25_PbI_3_ PeNPLs before and after 7 days of ambient aging.


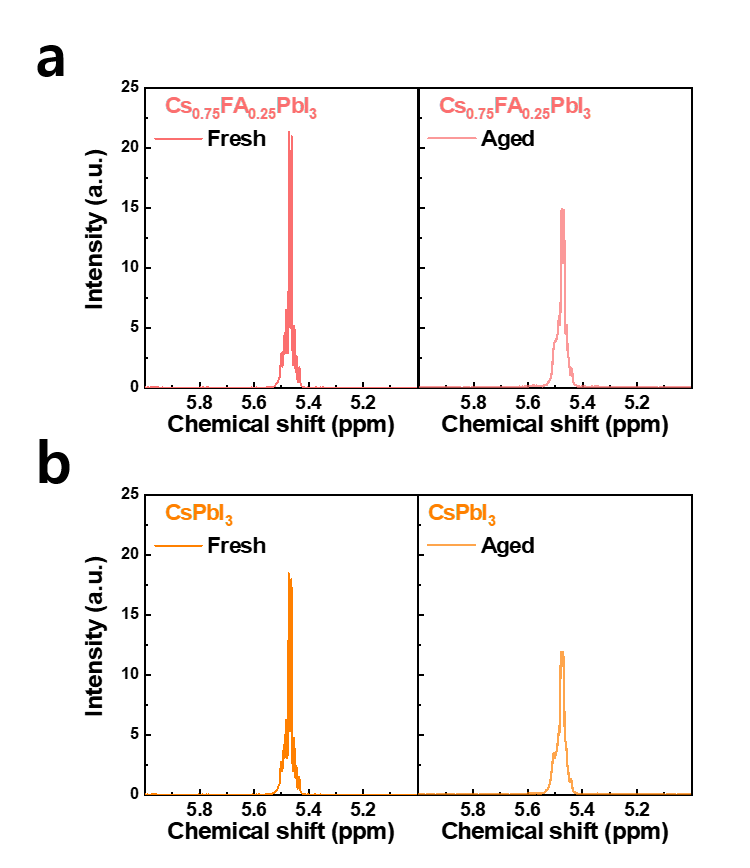


**Figure S11**. ^1^H-NMR spectra of **a** CsPbI_3_ and **b** Cs_0.75_FA_0.25_PbI_3_ PeNPLs before and after 7 days of ambient aging.

**
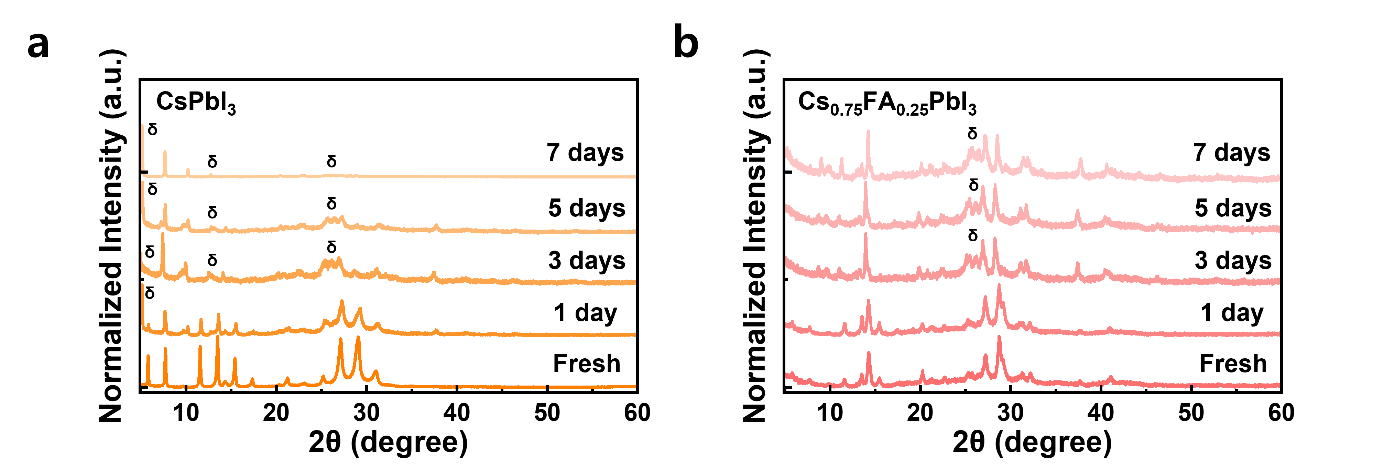
**

**Figure S12.** Change in the X-ray diffraction patterns of **a** CsPbI_3_ and **b** Cs_0.75_FA_0.25_PbI_3_ PeNPL films over time after storage in ambient conditions (40% relative humidity, 20 °C, stored in the dark).


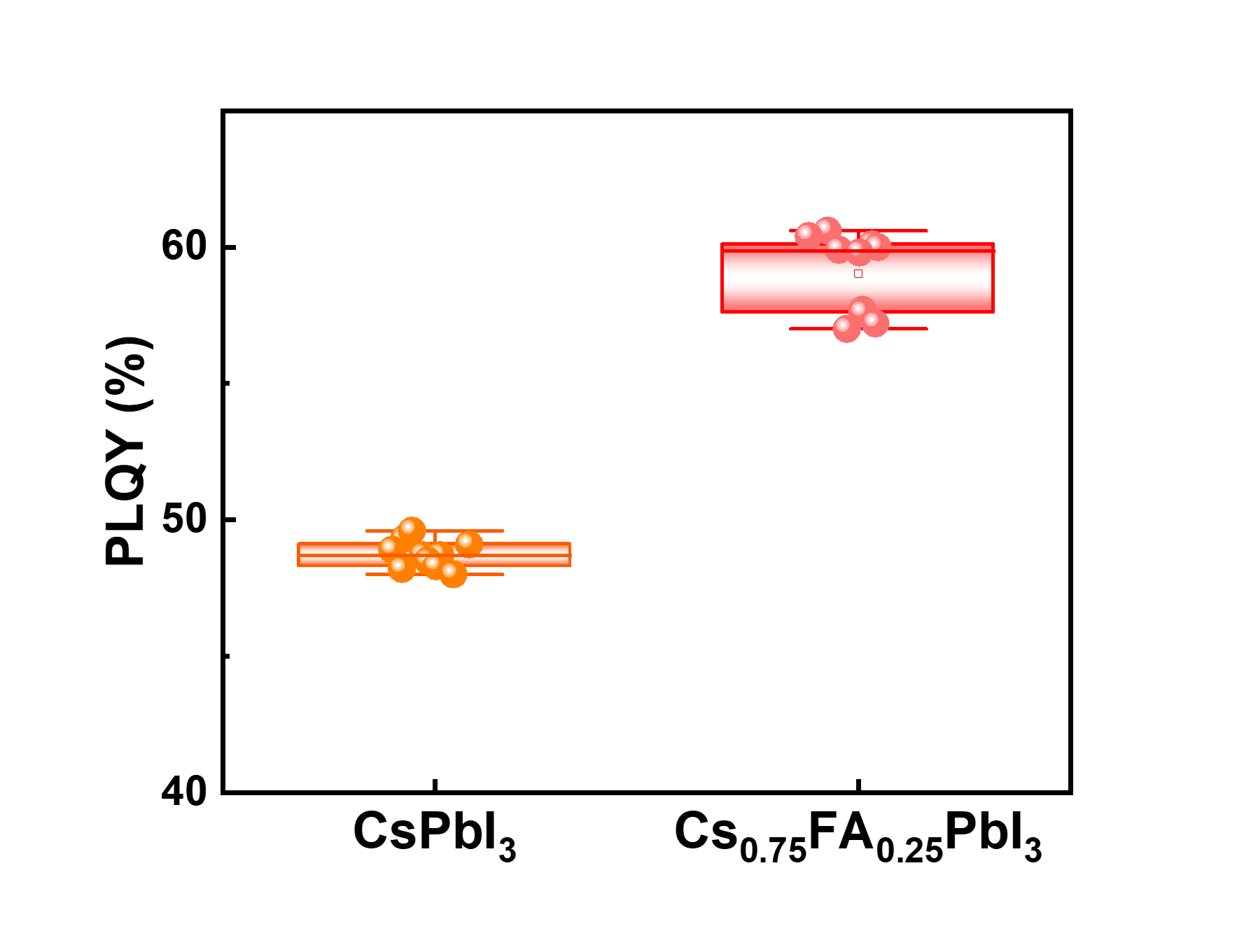


**Figure S13**. PLQY distributions of colloidal PeNPLs. Samples were measured using a Quantaurus-QY Absolute PL quantum yield spectrometer (HAMAMATSU).


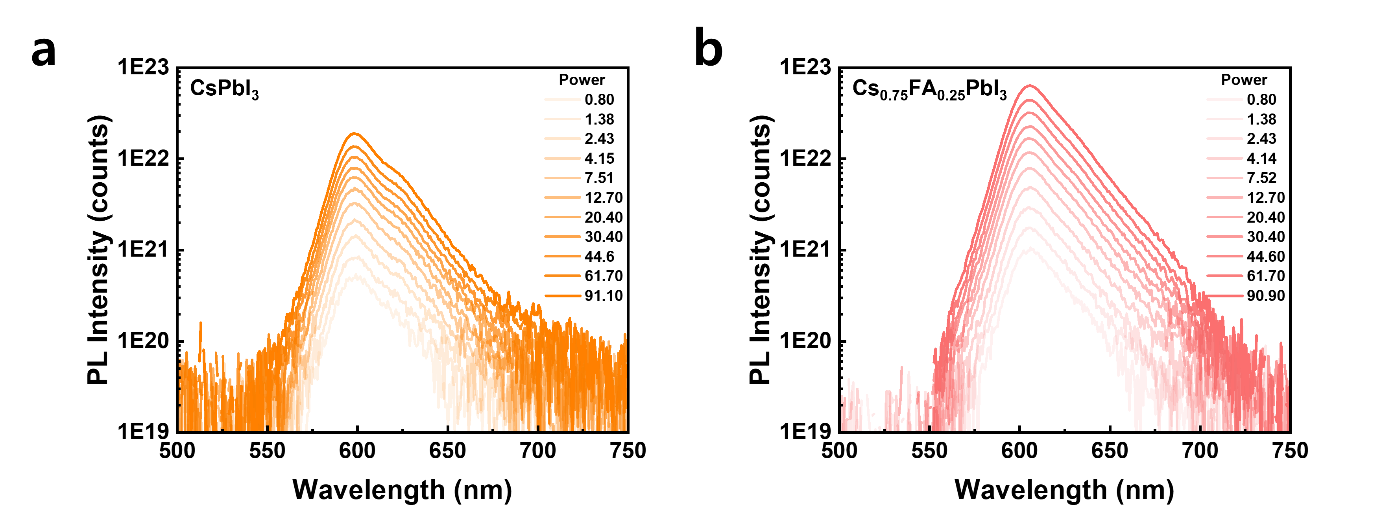


**Figure S14.** Change in the PL spectra of colloidal PeNPLs of **a** CsPbI_3_ and **b** Cs_0.75_FA_0.25_PbI_3_ depending on excitation power densities (in mW cm^-2^) from Fig. 4a in the main text. Samples were measured in ambient air, and excited with a 405 nm wavelength CW laser.


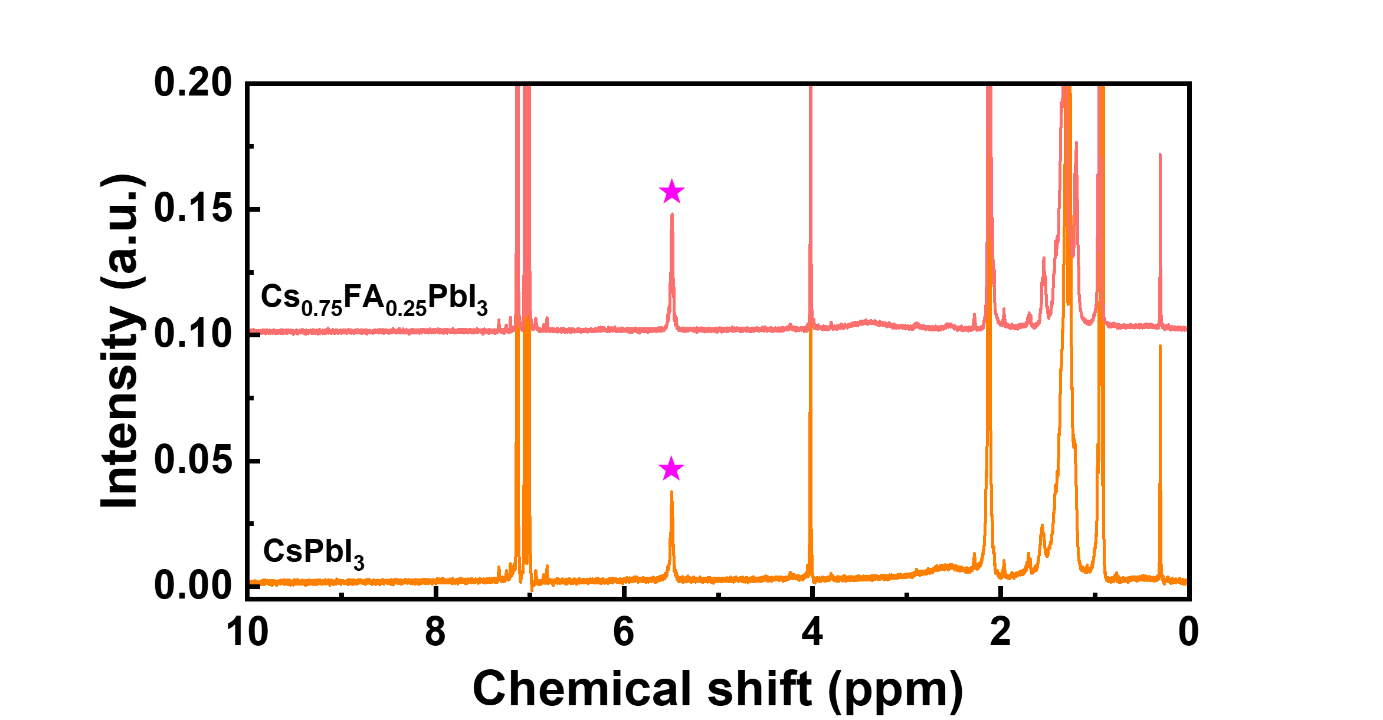


**Figure S15**. ^1^H-NMR spectra of colloidal PeNPL solutions in deuterated-toluene. Magenta stars indicate peaks corresponding to the oleic acid and oleylamine ligands.


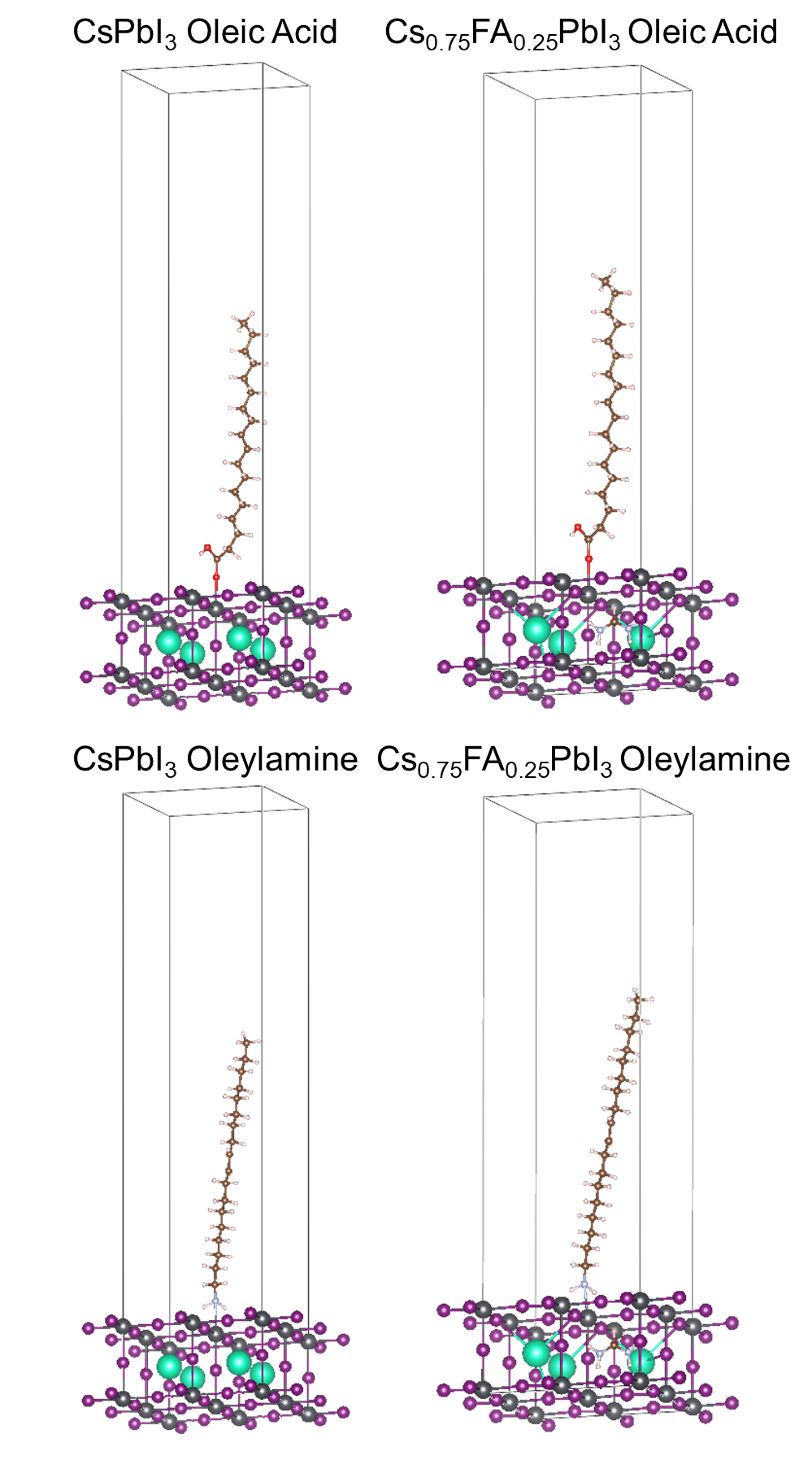


**Figure S16**. Calculation of the surface binding energy between the halide perovskite (CsPbI_3_ and Cs_0.75_FA_0.25_PbI_3_) and oleic acid and oleylamine ligand using electron localization functions from DFT.


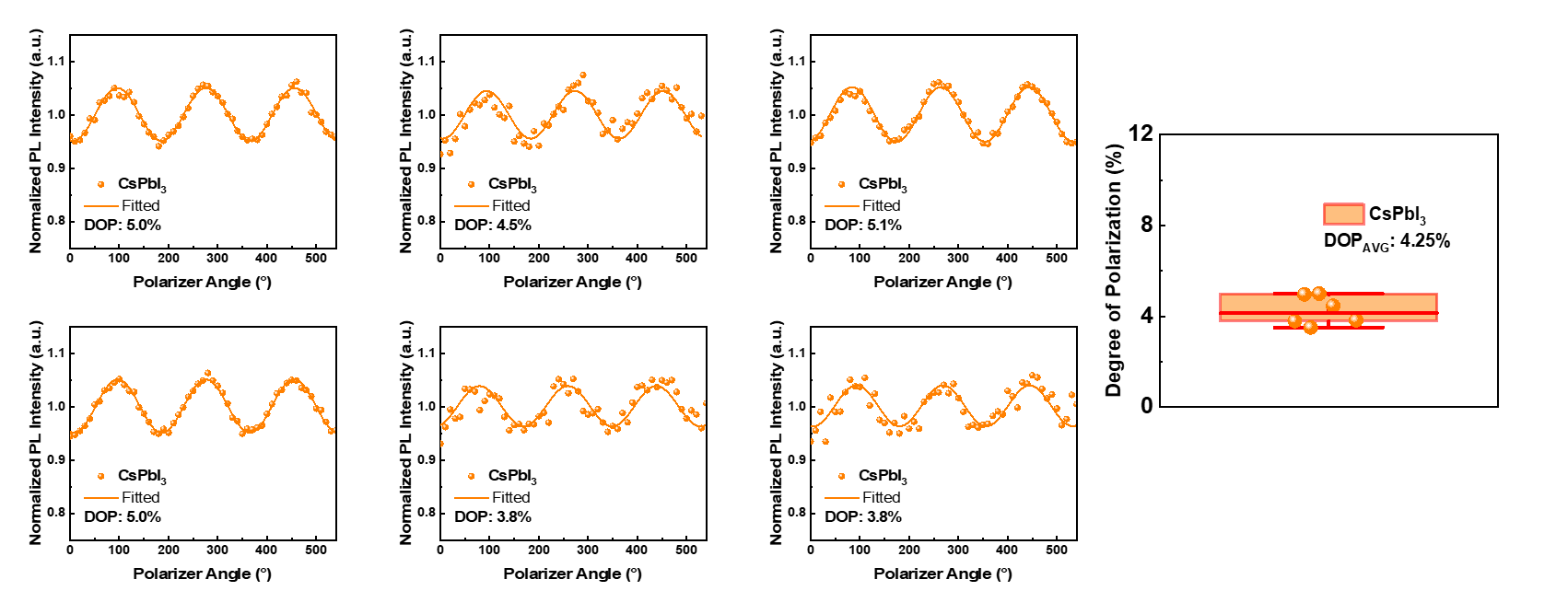


**Figure S17**. Polarization dependence of the normalized PL intensity of CsPbI_3_ PeNPLs films made in different batches, along with the distribution in the degree of linear polarization (DOP) determined from these measurements. Measurements performed inside a N_2_-filled glovebox with a 405 nm wavelength CW laser at 37.3 mW cm^-2^ power density.


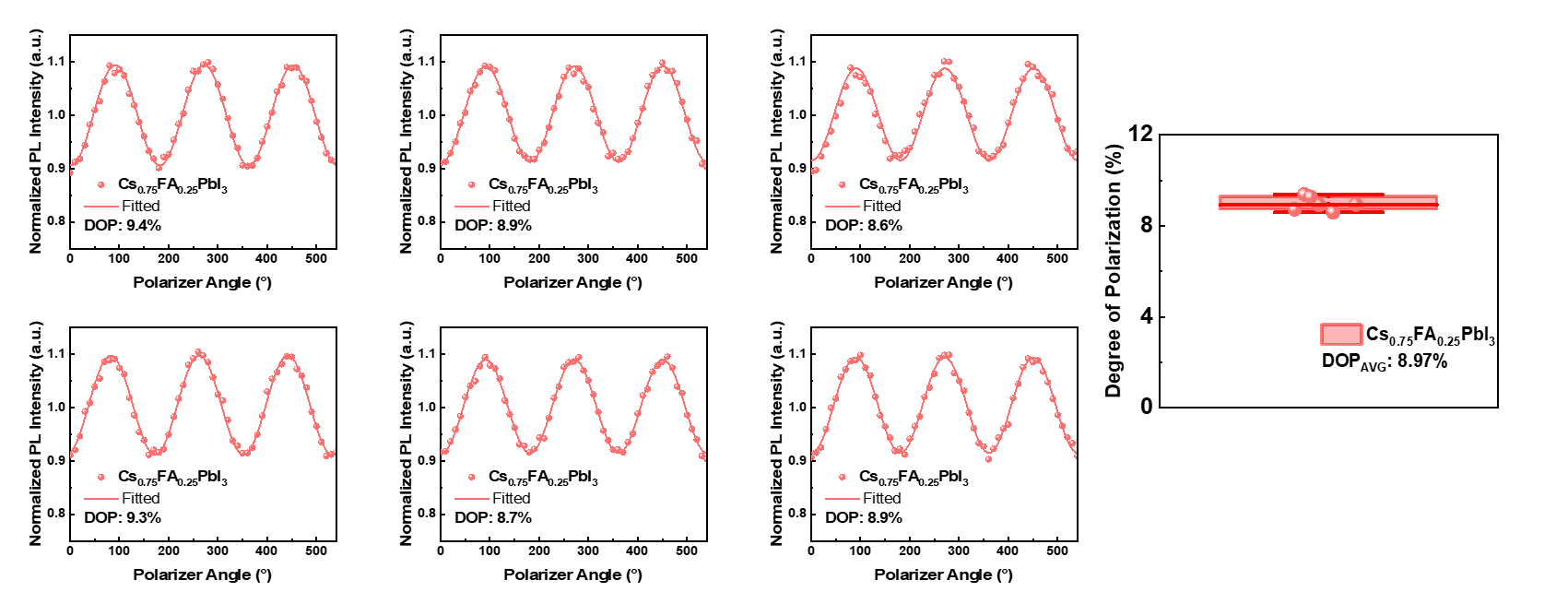


**Figure S18**. Polarization dependence of the normalized PL intensity of Cs_0.75_FA_0.25_PbI_3_ PeNPLs film made in different batches, along with the distribution in the degree of linear polarization (DOP) determined from these measurements. Measurements performed inside a N_2_-filled glovebox with a 405 nm wavelength CW laser at 37.3 mW cm^-2^ power density.


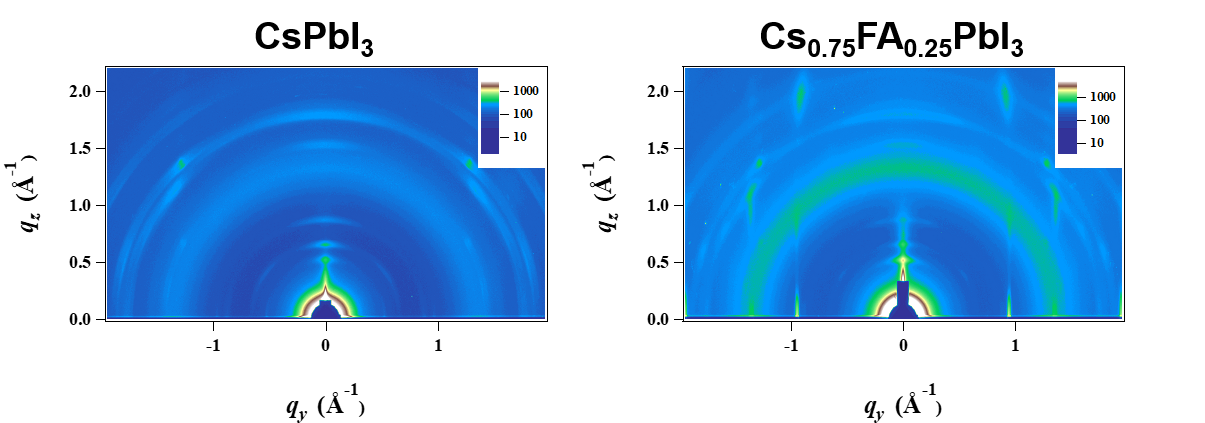


**Figure S19**. Grazing-incidence wide-angle X-ray scattering (GIWAXS) patterns of edge-up oriented CsPbI_3_ and Cs_0.75_FA_0.25_PbI_3_ PeNPL thin films. Sharper Bragg spots along *q*_z_ in the *x* = 0.25 PeNPL indicate improved vertical alignment of the PeNPL superlattice.

**Table S1**. XPS analysis of each PeNPLs.

| **Sample**  **configuration** | **Atomic %** | | | |
| --- | --- | --- | --- | --- |
|  | **Cs** | **Pb** | **I** | **N** |
| CsPbI_3_ | 0.89 | 1.00 | 4.02 | 1.62 |
| Aged-CsPbI_3_ | 0.45 | 1.00 | 2.35 | 1.10 |
| Cs_0.75_FA_0.25_PbI_3_ | 0.76 | 1.00 | 4.01 | 1.80 |
| Aged-Cs_0.75_FA_0.25_PbI_3_ | 0.61 | 1.00 | 3.12 | 1.46 |

**REFERENCES**

1 Lin, C.-C. *et al.* Exploring the Origin of Phase-Transformation Kinetics of CsPbI3 Perovskite Nanocrystals Based on Activation Energy Measurements. *The Journal of Physical Chemistry Letters* **11**, 3287-3293 (2020). https://doi.org/10.1021/acs.jpclett.0c00443
